# Multilayer network alignment based on topological assessment via embeddings

**DOI:** 10.1186/s12859-023-05508-5

**Published:** 2023-11-06

**Authors:** Pietro Cinaglia, Marianna Milano, Mario Cannataro

**Affiliations:** 1 Department of Health Sciences, Magna Graecia University, 88100 Catanzaro, Italy; 2 Department of Experimental and Clinical Medicine, Magna Graecia University, 88100 Catanzaro, Italy; 3 Data Analytics Research Center, Department of Medical and Surgical Sciences, Magna Graecia University, 88100 Catanzaro, Italy

**Keywords:** Multilayer networks, Network Alignment, Network analysis, Embeddings, Topological similarity

## Abstract

**Background:**

Network graphs allow modelling the real world objects in terms of interactions. In a multilayer network, the interactions are distributed over layers (i.e., intralayer and interlayer edges). Network alignment (NA) is a methodology that allows mapping nodes between two or multiple given networks, by preserving topologically similar regions. For instance, NA can be applied to transfer knowledge from one biological species to another. In this paper, we present *DANTEml*, a software tool for the Pairwise Global NA (PGNA) of multilayer networks, based on topological assessment. It builds its own similarity matrix by processing the node embeddings computed from two multilayer networks of interest, to evaluate their topological similarities. The proposed solution can be used via a user-friendly command line interface, also having a built-in guided mode (step-by-step) for defining input parameters.

**Results:**

We investigated the performance of *DANTEml* based on (i) performance evaluation on synthetic multilayer networks, (ii) statistical assessment of the resulting alignments, and (iii) alignment of real multilayer networks. *DANTEml* over performed a method that does not consider the distribution of nodes and edges over multiple layers by 1193.62%, and a method for temporal NA by 25.88%; we also performed the statistical assessment, which corroborates the significance of its own node mappings. In addition, we tested the proposed solution by using a real multilayer network in presence of several levels of noise, in accordance with the same outcome pursued for the NA on our dataset of synthetic networks. In this case, the improvement is even more evident: +4008.75% and +111.72%, compared to a method that does not consider the distribution of nodes and edges over multiple layers and a method for temporal NA, respectively.

**Conclusions:**

*DANTEml* is a software tool for the PGNA of multilayer networks based on topological assessment, that is able to provide effective alignments both on synthetic and real multi layer networks, of which node mappings can be validated statistically. Our experimentation reported a high degree of reliability and effectiveness for the proposed solution.

## Background

Network graphs (or simply networks) allow modelling the real world objects in terms of their relationships, by visualizing how the objects (nodes) are interconnected (edges) with each other. To give an example, these allow investigating topological and biological hypotheses based on the interactions existing between biological objects, by applying techniques of representation learning, clustering, or data mining [1, 2].

A multilayer network is an effective choice when several types of entities need to coexist in the same model, and their own connections are modelled. It is modelled a set of nodes, edges, and layers, where the interpretation of the layers depends on the implementation of the model, that may be homogeneous or heterogeneous based on the types of entity represented by the nodes [3]. Furthermore, it supports both intralayer and interlayer edges. The former is fully similar to the concept of edge described for classic networks, the second one instead represents a connection between nodes included in different layers.

Within this context, the relationship between temporal networks and multilayer networks should be mentioned. A temporal network (or time-varying network, or by extension dynamic network) [4] allows modelling a structure composed of the same type of entities, whose topology evolve over time [5, 6]. It can be defined in terms of a multilayer network [7], that includes temporal information on the edges. Therefore, we may consider the temporal networks as a particular case of multilayer networks, where the distribution does not occur in time spans but on layers of interest. Therefore, temporal networks (or time-varying networks) were included among the classes of multilayer networks [8], where each layer encodes the same type of interaction at different time points; briefly, each time point is a layer, representing objects in the same evolutionary time-window.

This assumption allowed us to extend our own existing solution for the alignment of dynamic networks (i.e., *DANTE* [9]), to support the alignment of multilayer networks.

Reflecting how described in the context of biological system, the multilayer networks allow properly modelling the interconnected units in a structure able to properly represent the evolutionary and heterogeneous nature of these, by interconnecting even more than one set of biological objects.

Network Alignment (NA) compares networks by finding a node mapping that conserves topologically similar regions from these. Therefore, the alignment of two given networks (i.e., source and target networks) produces a set of aligned node pairs, where a node of the first network is mapped to one of the second one [10]. In the Local NA (LNA), we will look to the matches between local regions, admitting many-to-many ones. In the Global NA (GNA), we will look to the best overlapping between the whole topologies of interests, only admitting one-to-one matches [11].

NA is a technique used to transfer knowledge from a network to another having a more complex topology. To give a non-exhaustive example, some genes in rat or mouse share functions with the genes in humans. Therefore, NA may be used to transfer this information, to simplify the in-vivo genotyping, as well as in vitro studies, by assuming that the similarities between the interaction in the networks of two species correspond to similarities in biological processes.

The scope of multilayer networks is still in an early stage, so that the literature does not report solutions specifically designed for the Pairwise GNA (PGNA) of multilayer networks. Nowadays, the only solution supporting the NA of multilayer networks has been proposed by Milano et al. [12] (i.e., *MultiLoAl*). However, it exploits a LNA heuristic, based on a set of seed nodes and the homology of the given networks.

The existing methodologies for PGNA do not properly process the multilayer networks, having been developed for other network’s models. For completeness, we propose an overview of the well-known ones, by focusing on those involved in our experimentation.

*MAGNA++* [13] is perhaps the most used tool for comparative purposes in the field of PGNA of static networks. It works by applying a genetic approach for maximizing node and edge conservation over successive permutations. Similarly, *DynaMAGNA++* [14] performs the PGNA of dynamic networks, based on event duration-based representation. Briefly, it handles a dynamic network as a set of static networks, each of which represents a specific time point. The evolution over time is maintained by evaluating the changes between the topologies of successive time points, by applying an extension of Graphlet Degree Vector (GDV) [15] for dynamic networks. GDV is also used by *DynaWAVE* for node conservation, otherwise, it performs the alignment via a greedy seed-and-extend strategy. According to the authors, this different approach is less accurate but faster than *DynaMAGNA++*.

In this paper, we present *DANTEml* (*DANTE for MultiLayer networks*), a novel software tool for the PGNA of multilayer networks, that uses topological assessment to build its own similarity matrix. As discussed, it is based on *DANTE* (see “Section DANTE: an algorithm for aligning dynamic networks” for detailed information), our existing solution for the PGNA of dynamic networks. Just like its progenitor, *DANTEml* builds the similarity matrix, on which the alignment function is performed, by analysing the embeddings computed for all nodes.

Our contribution is providing a ready-to-use tool, purely dedicated to the PGNA of multilayer networks, since the literature only reports methodologies applicable to individual sub-categories (e.g., temporal networks, and heterogeneous networks).

Finally, the main features of *DANTEml* are summarized as follows:

- computing the similarities between two multilayer networks of interest, based on node embeddings, in order to reflect their topological similarities;
- performing the GNA between pairs of multilayer networks;
- providing a user-friendly Command Line Interface, usable also by non-human operators for automating data processing, e.g., via scripting or pipelines.

## Design and Implementation of *DANTEml*

In this section, we present the design and implementation of *DANTEml*, by also focusing on the methods applied for processing, as well as on its use. Before dealing in details, let we firstly introduce how its own progenitor (*DANTE*) works for aligning pairs of dynamic networks, and how a multilayer network graph may be formally modelled.

### *DANTE*: an algorithm for aligning dynamic networks

Let us denote the source dynamic network by *G*(*V*, *E*1), with $V = \{v_1,v_2,\ldots ,v_n\}$ (*n* the number of nodes in *G*), and the target dynamic network by *H*(*U*, *E*2), with $U = \{u_1,u_2,\ldots ,u_m\}$ (*m* the number of nodes in *H*); we assumed without loss of generality: ${|}V{|} \le {|}U{|}$. *E*1 and *E*2 are the events (i.e., temporal edges) of *G* and *H*, respectively.

*DANTE* performs the PGNA based on three main steps: (i) evaluating the node features for each dynamic network (i.e., temporal embedding), (ii) constructing the similarity matrix, and (iii) performing the one-to-one node mapping between the source and the target.

The temporal embedding is induced by applying the Skip-Gram (SG) model [16] over the time points, iteratively. In the similarity matrix, the values are obtained by computing the cosine similarity between the pairs of vector embeddings, belonging to a node of the source network and a node of the target one, respectively.

*DANTE* performs the one-to-one node mapping (*f*) between *G* and *H*, by maximizing the objective function ($\phi$) between all pairs of nodes. Briefly, it aligns a node of *G* with a node of *H*, such that no node of *G* maps more than one node of *H*, and vice versa.

The alignment will provide a set of aligned nodes based on best match among all vectors, by handling the collisions via the global maximization of *f*.

The mapping $f: V \rightarrow U$ was implemented by adopting an iterative process, so that it produces a set of aligned node pairs (*v*, *f*(*v*)), with $v \in V$.

Formally, *f* is computed as follows:

1 $$\begin{aligned} f(v):= \{ u \mid \underset{u \in U}{argmax}\ \; \phi (v,u) \} \end{aligned}$$

where the objective function $\phi$ is defined as:

2 $$\begin{aligned} \phi \leftarrow cosine\_similarity(v,u): \{ \not \exists \phi (v',f(v')) \ge \phi (v,u), v' \in V \} \end{aligned}$$

### The Graph Model of a Multilayer Network

Formally, a multilayer network graph model $G_M$ can be described as $G_{M} = (V_M, E_M)$ [17], where $V_M$ and $E_M$ are a set of nodes and edges, respectively.

Referring to $G_{M}$, let us denote a generic intralayer by $G_{a}$ and a generic interlayer by $G_{b}$, consisting of its own set of edges $E_a$ (i.e., intralayer edges) and $E_b$ (i.e., interlayer edges), respectively. Elaborating, we may formally describe these as follows:

3 $$\begin{aligned} G_{a}= & {} (V_M, E_a): E_a = {((u, \alpha ), (v, \beta )) \in E_M | \alpha = \beta } \end{aligned}$$

4 $$\begin{aligned} G_{b}= & {} (V_M, E_b): E_b = E_M / E_a \end{aligned}$$

with $\alpha$ and $\beta$ the arrays of elementary layers, and (*u*, *v*) a generic pair of nodes. Note that in the proposed model, edges are undirected.

The multilayer networks are given in input to *DANTEml* in accordance with the described model. The latter has been implemented through a data structure based on an attributed edge list. Formally, we will have a set of tuples (*u*, *v*, *l*), such that *u* and *v* are the pair of nodes, and *l* is the attribute reporting the layer on which this edge insists; each intralayer is also defined by its own identifier.

This approach allows tracing it back to the whole topology by only considering the order of assignment of the identifiers (e.g., increasing integers), in order to reconstruct the multilayer network without loss of information. To give a non-exhaustive example of a multilayer network consisting of three layers, we can assign the identifiers 1, 2, and 3, for the first layer, the second one, and the interlayer, respectively.

**Fig. 1:**
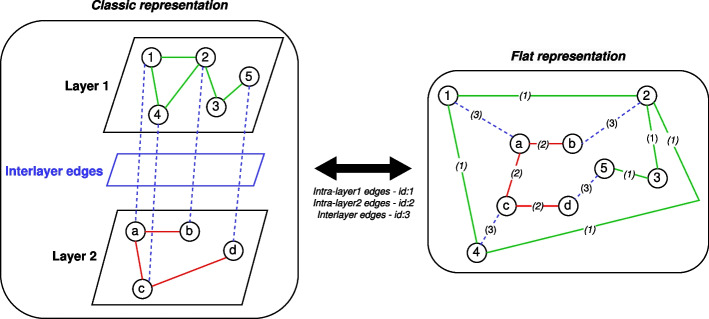
The figure shows a non-exhaustive toy example for a multilayer network consisting of two layers. The left side of the figure shows the classic representation of a multilayer networks, while the right side uses the (equivalent) flat representation. The intralayer edges belonging to the first layer are depicted in green and the ones for the second layer in red, while the interlayer edges in blue. The edges were tagged by using a dedicated identifier (id), as reported into the figure, in order to keep the whole topology intact, without losing information

In addition, a multilayer network can be defined by using an equivalent flat representation, that allows collapsing both intralayer and interlayer into a network graph model where edges and nodes were typed according to what they represented in the multilayer one; a non-exhaustive toy example consisting of two layers is shown in Fig. 1.

### DANTEml

*DANTEml* performs the alignment by inheriting the definition of *f* and $\phi$ from *DANTE*; we are referring to Eqs. 1 and 2, respectively. What differentiates *DANTEml* from *DANTE* mainly concerns the construction of the similarity matrix, to which *f* is applied. Even in this case, it is constructed based on a set of node embeddings, however, it does not consider a temporal evolution of each node, but the interactions of the latter over independent layers.

In a temporal network, the interlayers represented the edges conserved between one time point and the next one, and could be incorporated into the identity of the node that owned them, as an evolution of its own interactions. Otherwise, in this multilayer context, the interlayers link different entities, therefore, they can be treated as independent edges (i.e., no evolutionary) which has the only particularity of being outside the existence layer of the node that owns it.

In this novel context, a generic node is featured on the whole set of its own interactions (i.e., edges), that may also link different layers. This means that the characterization of a node considers all layers, through the analysis of both the intralayer and interlayer edges, in order to evaluate the relationship of each node with the overall topology of the multilayer network. This issue has required a redesign of the way to evaluate the features of the nodes from a topological point of view, in order to build the similarity matrix that is functional for a given pair of multilayer networks.

We have used the SG model via *node2vec* [18], for the representational learning on the multilayer network graph models in flat representation, to compute the node embeddings. It consists of a simple neural network based on one hidden layer, firstly designed for *word2vec* [16] and subsequently extended by *node2vec*.

Let us denote the number of neurons of the hidden-layer by *N*, the node embedding is a matrix with a dimension of $V \times N$ obtained by the dot product between the input and the hidden layer. The output layer consists of the dot product between each vector in the embedding matrix (*V* vectors) and its output vector (size equals to *N*), by producing a weighted matrix with size $N \times V$. For each iteration, the SG model selects a target node over a rolling window (*w*), of which the size represents the context location (*c*) at which the node is predicted. To give an example, the model will evaluate the nodes at $c-1$ and $c+1$, for $w=1$.

Briefly, *node2vec* learns the features of a given node by performing a fixed amount of random walks starting from this one, to explore its context and generate a random sample that can be embedded by using the SG model; the resulting output is a vector representation of the topological properties of the node in the network (i.e., node embedding).

*DANTEml* computes node embeddings for source and target multilayer networks, independently. These two sets of vector embeddings were correlated by calculating the similarities between all possible pairs, made up of a vector from the first set and one from the second one, respectively. In detail, *DANTEml* calculates the *cosine similarity* between a simple mean of the projection weight vectors of the given node in the source network, and the vectors for each node in the target network. The resulting similarities constitute the similarity matrix (*S*). From a point of view of the data structures used for the implementation of our solution, it consists of nested vectors.

Once the similarity matrix has been calculated, *DANTEml* applies *f* to *S*, for computing a preliminary (*pre*) one-to-one node mapping (or pre-mapping). Based on the latter, an iterative approach based on successive permutations was applied to maximize the Node Correctness (NC).

It is important to underline that the similarity matrix can also be acquired from external sources. The latter case is particularly advantageous for further improving the alignment score, on the basis of similarities between biological objects corroborated by the literature. To give an example, if the similarity between two genes exists, it can be used in the alignment step regardless of the topological location of the two genes in the respective networks or layers. Briefly, in this case, the embeddings are performed on the basis of the given similarity matrix, bypassing the in-depth evaluation of the networks through random walks.

Each permutation evolves the pre-mapping by searching alternative pairs within the similarity matrix that, even if with a lower similarity between the embeddings related to the nodes of interest, however these have a greater similarity in terms of global overlapping into its own layer with other candidate nodes.

Let us denote $(u_1,v_1)$ as a generic pre-mapping between the node $u_1$ and $v_1$, belonging to the source multilayer network and the target one, respectively. In our approach, the optimization of $u_1,v_1$ means searching a node $v_2$, such that the ratio between the similarity of the embeddings and the overlapping of nodes is globally the highest. For evaluating an overlapping, we opted for a simple and well-known metric in the field of node similarity: the Jaccard coefficient (Jc) [19]. We formally describe Jc in Eq. 5.

5 $$\begin{aligned} Jc = \frac{|\Gamma (u) \cap \Gamma (v)|}{|\Gamma (u) \cup \Gamma (v)|} \end{aligned}$$

with *u* and *v* the two nodes of interest, and $\Gamma (u)$ the set of neighbours of *u*.

This approach is performed by iteratively applying *f* to the similarity matrix, for each pair of nodes that do not also have a Jc equal to 1 (i.e., the mappings that are not already overlapped); this allows subsequent refinements. If the similarity between the embeddings already reflects the maximum achievable, the pair does not need to be optimized further and is considered as final. Note that the iterative processing is performed until all pairs of node mapping are maximized, or to a permutation amount defined by the user (default: 50% of the size of the pre-mapping; in our experimentation, this threshold has never been reached, and it seemed reasonable to us to contain the running time in the worst case).

**Fig. 2:**
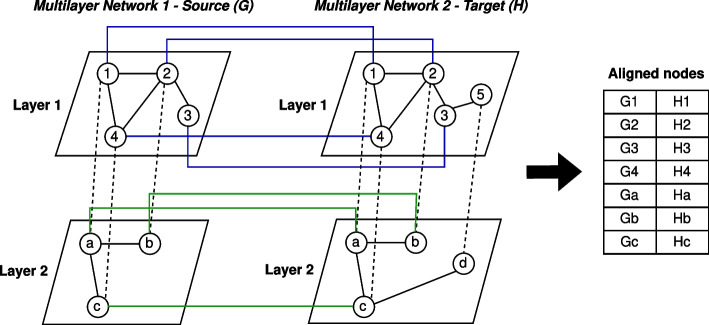
The figure shows a non-exhaustive toy PGNA between two multilayer networks having two layers, each one. The green and blue lines are the one-to-one node mappings between the nodes of the respective layer; in the figure, it was also reported as table

Summarizing, the PGNA between two generic networks produces a resulting one-to-one node mapping (see “Section Background”). For multilayer networks, the PGNA he must also evaluate the layered arrangement of the nodes, as well as the existence of the interlayer edges. Figure 2 shows a non-exhaustive toy alignment between two multilayer networks having two layers, each one. *DANTEml* produces an output in accordance with what has just been specified.

Finally, we also report below the main implementation details.

*DANTEml* was implemented in Python3, and it consists of three main functions related to (i) node embeddings, (ii) similarity matrix construction, and (iii) alignment, respectively.

The first allows for representational learning of a multilayer network graph model, based on the training of vector embeddings via random walks. It allows mapping a node to an embedding space based on its own topological features. The representational learning was processed by using a well-known Python package, i.e., *Gensim* [20], while the creation and manipulation of the network graph models was implemented through *NetworkX* [21]. Scientific computing (e.g., cosine similarity) and statistics were performed by using *Scipy* [22].

**Fig. 3:**
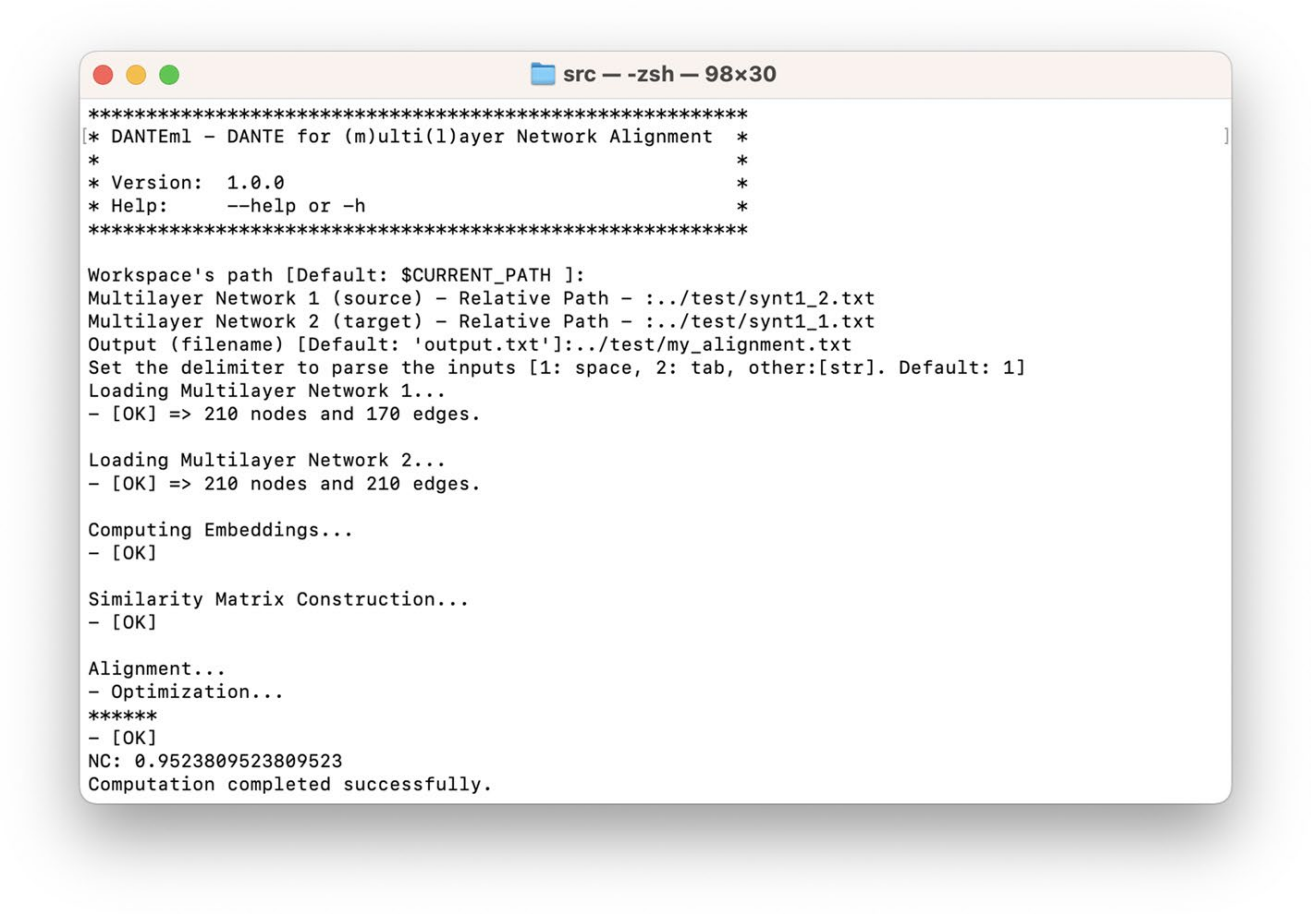
*DANTEml*’s screenshot: two synthetic multilayer networks were aligned. The figure shows the CLI, that allows you to set all parameters in guided mode; alternatively, these can be passed into the shell command

We have also implemented a user-friendly Command Line Interface (CLI) for human and non-human (e.g., script or pipeline), also having a built-in guided mode (step-by-step) for defining input parameters. *DANTEml*’s screenshot is shown in Fig. 3. Listing 1 briefly reports the cornerstones of using *DANTEml* through the help of the supported options and parameters.
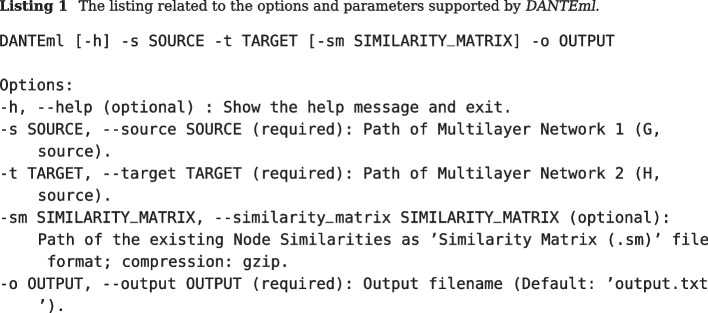

## Results

In this section, we report the results from our experimentation. However, first we will report the details of the datasets, and how these were obtained. Our experimentation focused on non-trivial case-studies consisting of heterogeneous entities.

### Datasets

#### Synthetic Multilayer Networks

Let us denote with *n* the number of nodes for a layer of interest, and with *m* the number of edges with which a new node attaches to existing nodes. Furthermore, let *p* be the probability to add a link to randomly chosen existing nodes between the *m* new edges defined for the layer of interest, and *q* be the probability that an edge insisting of a pair of nodes having at least another edge is removed (no node must be isolated) on the same layer. Note that when $p = q = 0$ this model behaves just like the Barabási-Albert model [23], a well-known random scale-free network model. The probabilities *p* and *q* can be used to simulate the duplication and divergence mechanisms that are detectable in a real biological network. For our experimentation, we generated 10 multilayer networks, by using the following two sets of arbitrarily parameters for each layer: (i) $[n=100, m=2, p=0.5, q=0.4]$ and (ii) $[n=100, m=2, p=0.3, q=0.7]$.

Furthermore, we based the model on 2 layers, with an amount of interlayer edges equal to 20% of the total interactions.

We also generated a set of four noisy versions, for each initial multilayer network, by removing $5\%$, $10\%$, $15\%$, $20\%$, and $25\%$ of randomly selected interactions from the whole set of intralayer and interlayer edges.

Briefly, the network pairs to be aligned consisted of the original multilayer network and all its own noisy versions.

#### Real Multilayer Network

We start by saying that datasets of real biological networks, downloadable and ready-to-use, are not available. Therefore, we modelled (*ad-hoc*) our own real multilayer network, by joining the following well-known datasets freely provided by Stanford Biomedical Network Dataset Collection (BioSNAP) [24]: Drug-Drug Interaction (DDI) network, Disease-Disease (DD) network, and Disease-Drug Association (DDA) network.

DDI and DD were used for modelling the two layers of our real network, while DDA for linking these through a set of interlayer edges. In post-processing, we improved the quality of the network by cleaning it from zero degree nodes, duplicate edges, and non-intersecting objects. The resulting network consisted of 8392 nodes and 128,200 edges, of which 72,809 were interlayer edges.

Similarly to the approach adopted for synthetic networks, we generated the noisy versions of this network. However, the noise was directly applied to the similarity matrix, by noising $5\%$, $10\%$, $15\%$, $20\%$, and $25\%$ of the similarities, both for diseases and drugs.

### Experimentation

We report the results from our experimentation. Specifically, we tested *DANTEml* based on (i) performance evaluation on synthetic multilayer networks, (ii) statistical assessment of the resulting alignments, and (iii) alignment of real multilayer networks. The information about the datasets is reported in “Section Datasets”.

For the synthetic networks, the similarity matrices were evaluated by *DANTEml*, while for the real network was used a similarity matrix, produced by simulating the real biological similarities between the entities that represent its nodes. In the latter case, we expect a better alignment score, despite the much larger size of the multilayer network.

#### Performance evaluation on synthetic dataset

 We aligned a set of synthetic networks with *DANTEml*, its own progenitor (*DANTE*), and a well-known method for the NA of static networks (i.e., *MAGNA++*), in order to compare its performance with a method optimized for dynamic/temporal networks and one that does not take into account the distribution of nodes and edges over multiple layers, respectively. The dataset was partially adapted both for its own progenitor (*DANTE*) and *MAGNA++*, in order to make parsable the networks (not being supported, officially): the former considered the layers as time points and the interlayer edges as temporal edges, the latter ignored the distribution of nodes and edges over multiple layers.

**Fig. 4:**
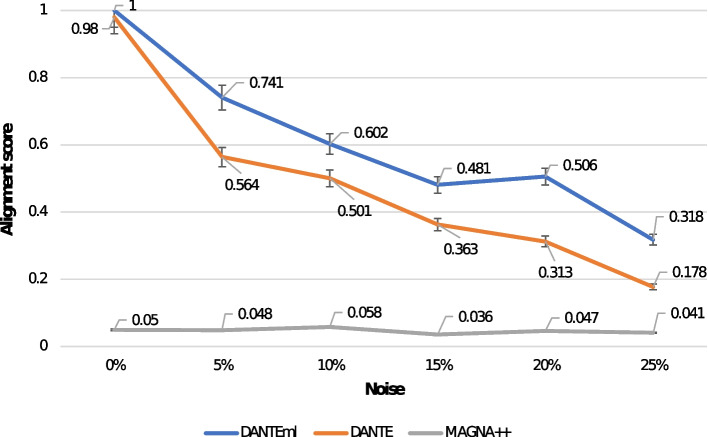
Comparison of the alignment scores, from a total of 50 alignments obtained by processing 10 synthetic multilayer networks and each of the noisy versions generated at $5\%$, $10\%$, $15\%$, $20\%$, and $25\%$

We addressed this outcome by performing 50 alignments with each of the mentioned software tools. It concerned the alignment between 10 synthetic multilayer networks and each of its noisy versions generated at $5\%$, $10\%$, $15\%$, $20\%$, and $25\%$. Figure 4 shows the related comparative representation of the alignments produced by each software tool of interest, in terms of alignment score. Furthermore, Table 1 reports the average of these, computed for all pairs from our dataset by *DANTEml*, *DANTE*, and *MAGNA++*, on the different level of noise; the overall mean across all groups is also reported.

**Table 1:** The table reports the average of the alignment scores, computed for all pairs from our dataset by *DANTEml*, *DANTE*, and *MAGNA++*, on the different level of noise; the overall mean across all groups is also reported

	Noise						Mean
	0%	5%	10%	15%	20%	25%	
DANTEml	1	0.741	0.602	0.481	0.506	0.318	0.608
DANTE	0.98	0.564	0.501	0.363	0.313	0.178	0.483
MAGNA++	0.05	0.048	0.058	0.036	0.047	0.041	0.047

**Fig. 5:**
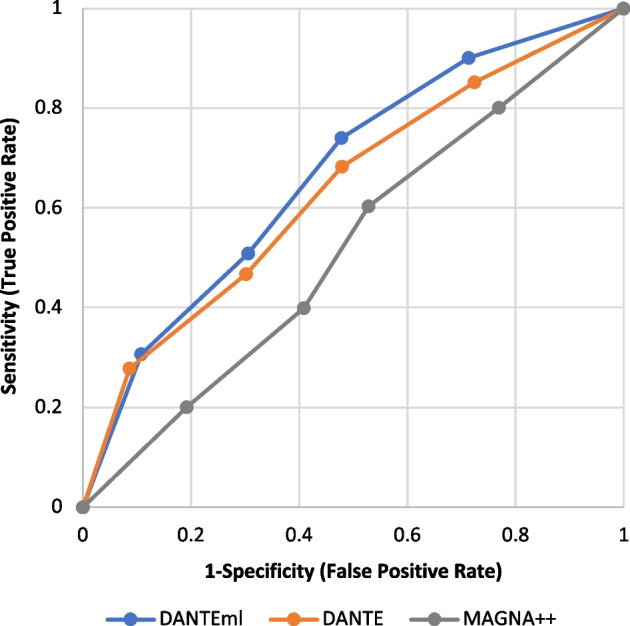
The figure shows the ROC curves related to the performance in terms of NC *DANTEml*, *DANTE*, and *MAGNA++*

We also evaluated the alignments in terms of True Positive Rate (TPR) and False Positive Rate (FPR), for plotting the ROC curves (see Fig. 5), as well as to calculate the related AUC (see Table 2). TPR is the *sensitivity*, while FPR is $1-specificity$.

**Table 2:** The table reports the AUC, F1-Score, and Matthews Correlation Coefficient (MCC), for *DANTEml*, *DANTE*, and *MAGNA++*

	AUC	F1-Score$_{avg}$	F1-Score$_{max}$	F1-Score$_{norm}$	MCC
DANTEml	0.760	0.571	0.872	0.65	0.541
DANTE	0.730	0.481	0.728	0.54	0.128
MAGNA++	0.621	0.070	0.103	0.080	-0.318

In details, F1-Score is shown through its average, maximum and normalized value: F1-Score$_{avg}$, F1-Score$_{max}$, and F1-Score$_{norm}$, respectively. The normalization is calculated on the noise level

#### Statistical assessment

 Foremost, we have proved the impossibility for a classical NA method (i.e., *MAGNA++*) to be able to produce statistically significant node mappings, between networks that are distributed over multiple layers (i.e., multilayer networks), in order to give more prominence to the fact that this issue is instead well processed by our solution. Tables 3 and 4 report the descriptive analysis and the One-Way ANOVA test [25], respectively, for *MAGNA++*. ANOVA is a well-known statistical approach for comparing several independent groups, by analysing the variances between and within these, in order to rank features as well as to classify the performance between groups [26]. Therefore, we considered the alignments at different noisy level, to constitute independent groups among which we could assume to exist a relationship due to degradation of the initial topology due to noise; alternatively, the node mappings may be considered as random output, and these should not be considered as valid alignments.

**Table 3:** Descriptive analysis related to alignment score, from the results produced by MAGNA++

Group	N	Mean	SD	SE	Coefficient of Variation
10	10	0.05804	0.04241	0.01341	0.73064
15	10	0.03564	0.03300	0.01043	0.92582
20	10	0.04705	0.02963	0.00937	0.62980
25	10	0.04134	0.03068	0.00970	0.74203
5	10	0.04847	0.04152	0.01313	0.85647

Groups were defined on the basis of noise

**Table 4:** One-Way ANOVA test, for the results produced by *MAGNA++*

Cases	Sum of Squares	df	Mean Square	F	p
Group	0.00281	4	0.00070	0.54665	0.70235
Residuals	0.05787	45	0.00129		

Groups were defined on the basis of noise. The *F*-ratio value is 0.54665, and the *p* value is 0.70235: the result is not significant at $p < 0.05$

Similarly, the same approach was applied to *DANTEml*. We corroborated the non-randomness of the node mappings computed by the latter. Tables 5 and 6 report the descriptive analysis and the One-Way ANOVA test, respectively, for *DANTEml*.

**Table 5:** Descriptive analysis related to the alignment score, from the results produced by *DANTEml*

Group	N	Mean	SD	SE	Coefficient of Variation
10	10	0.60244	0.17505	0.05536	0.29057
15	10	0.48106	0.13728	0.04341	0.28537
20	10	0.50591	0.16227	0.05132	0.32076
25	10	0.31792	0.12020	0.03801	0.37809
5	10	0.74094	0.20141	0.06369	0.27183

Groups were defined on the basis of noise

**Table 6:** One-Way ANOVA test, for the results produced by *DANTEml*

Cases	Sum of Squares	df	Mean Square	F	p
Group	0.97694	4	0.24424	9.33376	0.00001
Residuals	1.17751	45	0.02617		

Groups were defined on the basis of noise. The F-ratio (F) value is 9.33376, and the *p* value is 0.00001: the result is significant at $p < 0.05$

In addition, the statistical evaluation related to our solution was further explored by also performing a McNemar’s test on all pairwise alignments of our synthetic dataset, in accordance with Mohammadi et al. [27]. Generally, this approach is also indicated for predictive models. We have built a 2*x*2 contingency table from the average results produced by *DANTEml* (Case) in aligning all networks of our dataset (noisy ones included), and the best case (manually generated) that perfectly aligns all nodes (Control); data was normalized between 0 and 100. The contingency table (see Table 7) consisted of the following items (row–column indexes):

- *0–0* no. of pairs aligned correctly by both Case and Control: 61.
- *0–1* no. of pairs misaligned by Case, but not by Control: 8.
- *1–0* no. of pairs aligned correctly by Control, but not by Case: 31.
- *1–1* no. of pairs misaligned by both Case and Control: 0.

**Table 7:** McNemar’s test—Contingency table (Yes: aligned correctly, No: misaligned)

	Control		
Case	Yes	No	Total
Yes	61	8	69
No	31	0	31
Total	92	8	100

The two-tailed *p* value was 0.0004, calculated with McNemar’s test with the continuity correction.

The *p* value answered this issue: if there is no association between the node mapping processed by *DANTE* and the correctly aligned nodes, what is the probability of observing such a large discrepancy (or larger) between the number of the two kinds of discordant pairs? A small *p* value is evidence that there is an association between the node mapping processed by *DANTE* and the correctly aligned nodes; by conventional criteria, it can be considered to be statistically significant for a value less than 0.05 ($p < 0.05$).

#### Alignment of real multilayer networks

 We evaluated the performance of *DANTEml* on a case-study based on real multilayer networks.

**Fig. 6:**
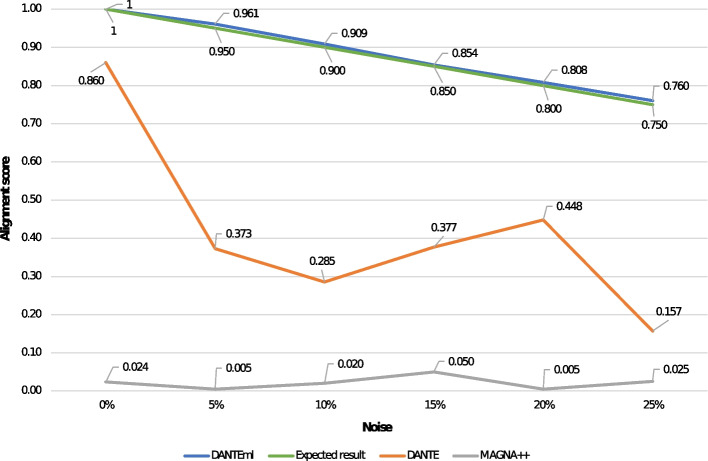
This figure shows the scores computed by aligning a real multilayer networks with itself, and noising the given similarity matrix by $5\%$, $10\%$, $15\%$, $20\%$, and $25\%$, that *DANTEml* used for node mapping. A result greater than the expected one is better. In addition, we included the alignments produced by *DANTE* and *MAGNA++* on the same dataset

We computed the alignments by pairing the network with itself, and by directly noising the given similarity matrix by $5\%$, $10\%$, $15\%$, $20\%$, and $25\%$, that *DANTEml* used for node mapping. This approach allowed us to be able to estimate what the expected alignment score could be: since the noise is directly applied to a network based on real entities (i.e., diseases and drugs), the true node mapping will have to refer to the applied noise, any higher scores are possible as the proposed solution evaluates the network topologies, and it could recover noisy structures. Figure 6 shows the results. The latter also shows the alignments produced by *DANTE* and *MAGNA++* on the same dataset. We calculated an average value of 0.88, 0.42, 0.02 for *DANTEml*, *DANTE*, and *MAGNA++*, respectively; the expected result was 0.88, on average.

## Discussion

We evaluated the alignments produced by *DANTEml*, *DANTE*, and *MAGNA++*, via well-known KPI and statistical methods.

We had also initially chosen *DynaWAVE* and *DynaMAGNA++*, in that these are (with *MAGNA++*) well-known methods, generally used for comparison. Unfortunately, we were forced to exclude them. Both ones are based on DGDV, whose analysis algorithm has been implemented by using the node identifiers to link successive time points. Our dataset consists of multilayer networks that are made up of different nodes for the various layers, we hypothesized that it was this that caused infinite loops during the experimentation, making it impossible to use these methods, and leading us to exclude them.

According to Chen et al. [28], no accepted criteria are generally accepted in literature for evaluating alignment performance or comparing two methods. It is ambiguous to indicate a criterion as the best or gold-standard, however, the assessment of alignment performance through well-known Key Performance Indicators (KPIs) is globally accepted [29]. Therefore, we have evaluated the performance of *DANTEml* based on the following well-known KPIs: NC, Precision, Recall, F1-Score, the Area Under the Receiver Operating Characteristic (ROC) Curve (AUC, or AUROC) [30]. Furthermore, we calculated the Matthews Correlation Coefficient (MCC). It is equivalent to chi-square statistics for a 2*x*2 contingency table [31]. MCC is a value between − 1 and 1 to be interpreted as follows:

- perfect alignment (or true node mapping): +1;
- no valid information (or random mapping): 0;
- inconsistency between alignment and true node mapping: − 1.

NC evaluates the Precision of the alignment, by showing the ratio of aligned node pairs to the true node mapping. However, it is only defined for GNA, and no metric was specially designed for comparing GNA and LNA methods [32], therefore, therefore, we did not consider it appropriate, to compare *DANTEml* (global NA) and *MultiLoAl* (local NA), since the former produces a one-to-one node mapping, while the latter a many-to-many node mapping.

In our experimentation, we reported NC by using its own unity-based normalisation via feature scaling, as alignment score, while Precision and Recall have been combined in F1-Score, based on their harmonic mean. Please note that the only mentioned solution specially designed for the alignment of multilayer networks (i.e., *MultiLoAl*, see “Section Background”) applies a heuristic for the local alignment, that needs of a set of seed nodes to evaluate a similarity based on their homology, not on direct topological analysis. Therefore, *MultiLoAl* needs the whole set of node pairs that can be considered as perfect matches between the two networks; this approach simulates the biological similarities, e.g., between genes, or the relationships between genes and proteins.

Briefly, we have constructed the pairs to be aligned by defining a set of initial multilayer networks that was aligned with its own noisy counterparts to increasing noise levels. Therefore, we expected nodes to be coupled with their counterparts (i.e., TP), and that as noise increases, the accuracy of the alignment degrades while FP increase. The latter is a physiological result of the fact that as the noise increases, the noisy counterpart varies its topology in a non-negligible way and some node may become unrecognizable. In fact, it doesn’t represent a real error, so much so that we used this test to measure to what degree of noise the matches can still be considered optimal for our solution. Based on the above considerations, a True Negative (TN) and False Negative (FN) are a truly non-existent match and a missing match, respectively.

We investigated the performance of DANTEml based on (i) performance evaluation on synthetic multilayer networks, (ii) statistical assessment of the resulting alignments, and (iii) alignment of real multilayer networks.

The first test demonstrates the efficiency of *DANTEml* compared to a method that does not consider the distribution of nodes and edges over multiple layers, as well as the improvements apported in comparison with a method only optimized for a specific category of multilayer networks (i.e., temporal networks). Results shows *DANTEml* over performed the former by $1193.62\%$ and the latter by $25.88\%$, in terms of alignment score evaluated on all dataset (see *Mean* of Table 1).

According to Nahm et al. [30], the interpretation of AUC shows a good performance for *DANTEml* and *DANTE*, while it shows a poor one for *MAGNA++* (see Table 2). F1-Scores are in accordance with the AUC values, by confirming the goodness. The poor results produced by *MAGNA++* were expected, in that it does not (rightly) take into account the distribution of nodes and edges between multiple layers. Likewise, we had also already supposed that *DANTE* would produce valid results, being in any case capable of managing dynamic networks. However, what we must take into consideration is how *DANTEml* manages to make a significant contribution, both with respect to cases that are not perfectly specific, and to the traditional ones for simple static networks.

*DANTEml* showed a strong positive relationship between the computed alignment and the true node mapping, while *DANTE* a negligible relationship. According to our statistical assessment, *MAGNA++* showed an inconsistent result that should be discarded.

The statistical assessment was performed via One-Way ANOVA test, and McNemar’s test, as explained in “Section Results”.

We demonstrated the impossibility for a classical NA method to produce statistically significant alignments; already highlighted by the MCC related to *MAGNA++*.

In addition, we demonstrated the statistical significance of the alignments produced by *DANTEml*. In addition, we evidenced that there was an association between the node mapping processed by *DANTE* and the correctly aligned nodes, by also corroborating the non-randomness of the node mappings computed by the proposed solution via McNemar’s test.

The results validated the hypothesis above; see Tables 4 and 6 for One-Way ANOVA test, while for McNemar’s test the *p* value is 0.0001. By conventional criteria, the *p* values are statistically significant ($p < 0.05$).

Finally, our method produced effective alignments, whose quality is statistically correlated to the topological similarity existing between the pairs of given networks.

In the third test (see Fig. 6), we pursued the same outcome of NA of synthetic networks, by aligning a real multilayer network with itself, based on the noised similarity matrix ($5\%$, $10\%$, $15\%$, $20\%$, and $25\%$ noise).

We computed the expected alignment score from the true node mapping, to simulate a real case in which an optimal solution exists for the NA of multilayer networks. Note that higher scores are possible, as the proposed solution evaluates the network topologies, and it could recover noisy structures. The use of software tools for other network models (i.e., *DANTE*, and *MAGNA++*) are reported for illustrative purposes only. According to results (see Fig. 6), *DANTEml* shows an improvement of $+111.72\%$ and $+4008.75\%$ compared to *DANTE* and *MAGNA++*, respectively, while it is perfectly in line with the expected result. We hypothesize that this more marked difference, to the advantage of *DANTEml*, depended on the size of the network; much larger than synthetic ones, and consequently more distributed between the layers. The clear gap in the results demonstrates the clear advantage between a method that supports a model in which heterogeneous objects are distributed across multiple layers, and one that does not recognize this feature.

The resulting scores showed how the proposed solution is able to maintain a high degree of reliability and effectiveness also for the alignment between large networks built on the basis of real data. As discussed, an initial similarity matrix was used to simulate the real biological similarities between the entities that represent the nodes. The alignment scores were slightly better than the expected result, probably due to the successive permutations that are applied by *DANTEml* to its own pre-mapping to further maximize the node similarities, globally.

## Conclusions

In this paper, we present *DANTEml*, a software tool for the PGNA of multilayer networks based on topological assessment. It builds its own similarity matrix by processing the node embeddings computed from two given networks of interest.

Results showed that *DANTEml* over performed *MAGNA++* (a method that does not consider the distribution of nodes and edges over multiple layers) by $1193.62\%$, and *DANTE* (its own progenitor for temporal network) by $25.88\%$.

Furthermore, we performed the statistical assessment of the resulting alignments. By conventional criteria, the *p* values computed for our solution are statistically significant at $p < 0.05$; this corroborates the significance of its own node mappings. Briefly, *DANTEml* provided effective alignments of which node mappings were validated statistically. It has also been tested for aligning large multilayer networks based on real data, by showing a high degree of reliability and effectiveness. In this case, *DANTEml* showed an improvement of $+111.72\%$ and $+4008.75\%$ compared to *DANTE* and *MAGNA++*, respectively, as well as it was perfectly in line with the expected result.

Finally, *DANTEml* allowed aligning both synthetic and real multilayer networks, by proving itself an effective method, of which node mappings can be validated statistically.

### Availability and requirements

- Project name: DANTEml.
- Project home page: https://github.com/pietrocinaglia/danteml (accessed on 05 July 2023).
- Operating system(s): Platform independent.
- Programming language: Python 3.
- Other requirements: https://github.com/pietrocinaglia/danteml/blob/main/requirements.txt (accessed on 05 July 2023).
- Licence: the software is provided *AS IS* under MIT Licence.
- Any restrictions to use by non-academics: none.

## Data Availability

Additional details on installation and requirements, as well as example data, are available on the dedicated GitHub repository (https://github.com/pietrocinaglia/danteml, accessed on 05 July 2023).

## Abbreviations

NA: Network Alignment
GNA: Global Network Alignment
LNA: Local Network Alignment
PGNA: Pairwise Global Network Alignment
SG: Skip-Gram
Jc: Jaccard coefficient
CLI: Command Line Interface
KPI: Key Performance Indicator
NC: Node Correctness
AUROC (or AUC): Area Under the Receiver Operating Characteristic curve
TP: True Positive
FP: False Positive
TN: True Negative
FN: False Negative
TPR: True Positive Rate
FPR: False Positive Rate
SD: Standard Deviation
SE: Standard Error
df: Degrees of freedom

## References

[1] Hu L, Yang Y, Tang Z, He Y, Luo X (2023). FCAN-MOPSO: an improved fuzzy-based graph clustering algorithm for complex networks with multi-objective particle swarm optimization. IEEE Trans Fuzzy Syst.

[2] Zhao B-W, Su X-R, Hu P-W, Huang Y-A, You Z-H, Hu L (2023). iGRLDTI: an improved graph representation learning method for predicting drug-target interactions over heterogeneous biological information network. Bioinformatics.

[3] Hammoud Z, Kramer F (2020). Multilayer networks: aspects, implementations, and application in biomedicine. Big Data Anal.

[4] Cinaglia P, Cannataro M (2022). Network alignment and motif discovery in dynamic networks. Netw Model Anal Health Inform Bioinform.

[5] Chow K, Sarkar A, Elhesha R, Cinaglia P, Ay A, Kahveci T (2021). ANCA: alignment-based network construction algorithm. IEEE/ACM Trans Comput Biol Bioinform.

[6] Elhesha R, Sarkar A, Cinaglia P, Boucher C, Kahveci T. Co-evolving patterns in temporal networks of varying evolution. In: Proceedings of the 10th ACM international conference on bioinformatics, computational biology and health informatics. BCB ’19, 2019;pp. 494–503. ACM, New York. 10.1145/3307339.3342152.

[7] Thompson WH, Brantefors P, Fransson P (2017). From static to temporal network theory: applications to functional brain connectivity. Netw Neurosci.

[8] Lv Y, Huang S, Zhang T, Gao B (2021). Application of multilayer network models in bioinformatics. Front Genet.

[9] Cinaglia P, Cannataro M (2023). A method based on temporal embedding for the pairwise alignment of dynamic networks. Entropy.

[10] Vijayan V, Milenković T (2017). Aligning dynamic networks with DynaWAVE. Bioinformatics.

[11] Guzzi PH, Milenkovic T (2018). Survey of local and global biological network alignment: the need to reconcile the two sides of the same coin. Brief Bioinform.

[12] Milano M, Guzzi PH, Cannataro M (2022). Design and implementation of a new local alignment algorithm for multilayer networks. Entropy.

[13] Vijayan V, Saraph V, Milenković T (2015). MAGNA++: maximizing accuracy in global network alignment via both node and edge conservation. Bioinformatics.

[14] Vijayan V, Critchlow D, Milenkovic T (2017). Alignment of dynamic networks. Bioinformatics.

[15] Milenković T, Ng WL, Hayes W, Przulj N (2010). Optimal network alignment with graphlet degree vectors. Cancer Inform.

[16] Mikolov T, Sutskever I, Chen K, Corrado G, Dean J. Distributed representations of words and phrases and their compositionality. In: Proceedings of the 26th international conference on neural information processing systems—volume 2. NIPS’13, pp. 3111–3119. Curran Associates Inc., Red Hook, 2013.

[17] Kivelä M, Arenas A, Barthelemy M, Gleeson JP, Moreno Y, Porter MA (2014). Multilayer networks. J Complex Netw.

[18] Grover A, Leskovec J (2016). node2vec: Scalable Feature Learning for Networks. KDD.

[19] Liben-Nowell D, Kleinberg J. The link prediction problem for social networks. In: Proceedings of the twelfth international conference on information and knowledge management. CIKM ’03, pp. 556–559. Association for Computing Machinery, New York, NY, USA, 2003.

[20] Řehůřek R, Sojka P. Software framework for topic modelling with large corpora. In: Proceedings of the LREC 2010 workshop on new challenges for NLP frameworks, pp. 45–50. ELRA, Valletta, Malta 2010.

[21] Hagberg AA, Schult DA, Swart PJ. Exploring network structure, dynamics, and function using networkx. In: Varoquaux, G., Vaught, T., Millman, J. (eds.) Proceedings of the 7th Python in Science Conference, Pasadena, CA, USA, 2008; pp. 11–15.

[22] Virtanen P, Gommers R, Oliphant TE, Haberland M, Reddy T, Cournapeau D, Burovski E, Peterson P, Weckesser W, Bright J, van der Walt SJ, Brett M, Wilson J, Millman KJ, Mayorov N, Nelson ARJ, Jones E, Kern R, Larson E, Carey CJ, Polat İ, Feng Y, Moore EW, VanderPlas J, Laxalde D, Perktold J, Cimrman R, Henriksen I, Quintero EA, Harris CR, Archibald AM, Ribeiro AH, Pedregosa F, van Mulbregt P, SciPy 1.0 Contributors: SciPy 1.0: Fundamental Algorithms for Scientific Computing in Python. Nature Methods 2020;17:261–272 . 10.1038/s41592-019-0686-2. 10.1038/s41592-019-0686-2 PMC7056644 32015543

[23] Barabasi A-L, Oltvai ZN (2004). Network biology: understanding the cell’s functional organization. Nat Rev Genet.

[24] Zitnik M, Sosič R, Maheshwari S, Leskovec J. BioSNAP Datasets: Stanford Biomedical Network Dataset Collection. http://snap.stanford.edu/biodata. 2018.

[25] McHugh ML. Multiple comparison analysis testing in ANOVA. Biochemia Medica 203–209; 2011. 10.11613/bm.2011.029. 10.11613/bm.2011.029 22420233

[26] Owen RK, Cooper NJ, Quinn TJ, Lees R, Sutton AJ (2018). Network meta-analysis of diagnostic test accuracy studies identifies and ranks the optimal diagnostic tests and thresholds for health care policy and decision-making. J Clin Epidemiol.

[27] Mohammadi M, Atashin AA, Hofman W, Tan Y (2018). Comparison of ontology alignment systems across single matching task via the Mcnemar’s test. ACM Trans Knowl Discov Data.

[28] Chen B, Fan W, Liu J, Wu FX (2014). Identifying protein complexes and functional modules-from static PPI networks to dynamic PPI networks. Brief Bioinform.

[29] Zaslavskiy M, Bach F, Vert JP (2009). Global alignment of protein-protein interaction networks by graph matching methods. Bioinformatics.

[30] Nahm FS (2022). Receiver operating characteristic curve: overview and practical use for clinicians. Korean J Anesthesiol.

[31] Boughorbel S, Jarray F, El-Anbari M (2017). Optimal classifier for imbalanced data using Matthews correlation coefficient metric. PLoS ONE.

[32] Meng L, Striegel A, Milenković T (2016). Local versus global biological network alignment. Bioinformatics.

